# Estimating the Number of Heterosexual Persons in the United States to Calculate National Rates of HIV Infection

**DOI:** 10.1371/journal.pone.0133543

**Published:** 2015-07-27

**Authors:** Amy Lansky, Christopher Johnson, Emeka Oraka, Catlainn Sionean, M. Patricia Joyce, Elizabeth DiNenno, Nicole Crepaz

**Affiliations:** 1 Division of HIV/AIDS Prevention, National Center for HIV/AIDS, Viral Hepatitis, STD, and TB Prevention, Centers for Disease Control and Prevention, Atlanta, Georgia, United States of America; 2 ICF International at the Division of HIV/AIDS Prevention, National Center for HIV/AIDS, Viral Hepatitis, STD and TB Prevention, Centers for Disease Control and Prevention, Atlanta, Georgia, United States of America; Emory University's RSPH, UNITED STATES

## Abstract

**Background:**

This study estimated the proportions and numbers of heterosexuals in the United States (U.S.) to calculate rates of heterosexually acquired human immunodeficiency virus (HIV) infection. Quantifying the burden of disease can inform effective prevention planning and resource allocation.

**Methods:**

Heterosexuals were defined as males and females who ever had sex with an opposite-sex partner and excluded those with other HIV risks: persons who ever injected drugs and males who ever had sex with another man. We conducted meta-analysis using data from 3 national probability surveys that measured lifetime (ever) sexual activity and injection drug use among persons aged 15 years and older to estimate the proportion of heterosexuals in the United States population. We then applied the proportion of heterosexual persons to census data to produce population size estimates. National HIV infection rates among heterosexuals were calculated using surveillance data (cases attributable to heterosexual contact) in the numerators and the heterosexual population size estimates in the denominators.

**Results:**

Adult and adolescent heterosexuals comprised an estimated 86.7% (95% confidence interval: 84.1%-89.3%) of the U.S. population. The estimate for males was 84.1% (CI: 81.2%-86.9%) and for females was 89.4% (95% CI: 86.9%-91.8%). The HIV diagnosis rate for 2013 was 5.2 per 100,000 heterosexuals and the rate of persons living with diagnosed HIV infection in 2012was 104 per 100,000 heterosexuals aged 13 years or older. Rates of HIV infection were >20 times as high among black heterosexuals compared to white heterosexuals, indicating considerable disparity. Rates among heterosexual men demonstrated higher disparities than overall population rates for men.

**Conclusions:**

The best available data must be used to guide decision-making for HIV prevention. HIV rates among heterosexuals in the U.S. are important additions to cost effectiveness and other data used to make critical decisions about resources for prevention of HIV infection.

## Introduction

In the United States (U.S.), 25% of new HIV diagnoses in 2013 were attributable to heterosexual contact [1]. At the end of 2012, 26% of adults and adolescents living with diagnosed human immunodeficiency virus (HIV) infection in the United States had an infection attributable to heterosexual contact [1].

Although the Centers for Disease Control and Prevention (CDC) routinely uses population data from the Census Bureau to calculate HIV rates by selected demographic categories (e.g., sex, race/ethnicity, and age at diagnosis) no census data are available for HIV transmission categories (“risk groups”), and disease rate calculations require this number for the denominator. Recently, CDC used meta-analysis to estimate the proportion of the U.S. population in these risk groups, including men who have sex with men (MSM) [2] and persons who inject drugs (PWID) [3], and reported the population proportion who are men who have sex with men and inject drugs [4]. Population size estimates together with census and surveillance data were used to calculate disease rates among MSM and PWID. In this report we estimate the population proportion of heterosexuals and use it to calculate rates of heterosexually acquired HIV infection and rate ratios by sex, race/ethnicity, and age. Quantifying the burden of disease can inform effective prevention planning and resource allocation.

## Methods

Based on previous work developing HIV risk group population estimates [2–4], we identified three national probability surveys providing data on lifetime (ever) sexual activity to determine the proportion of the United States population classified as heterosexual. Data from the three surveys were combined using meta-analysis (S1 Table). We applied the proportion of heterosexual persons to census data to produce population size estimates. National HIV infection rates among heterosexuals were calculated using HIV surveillance data in the numerators (i.e., cases attributable to heterosexual contact) and the heterosexual population size estimates for the denominators. An analysis of “high-risk heterosexual” was conducted to estimate the upper bound prevalence estimate of heterosexually acquired HIV infection. Specific methods are detailed below.

### Definition of “Heterosexual”

Our definition of heterosexual was created to best correspond to the HIV transmission category used for surveillance [1] as our ultimate purpose for this analysis was to calculate disease rates. “Transmission category” is the HIV surveillance term for the classification of cases among those aged 13 years or older that summarizes a person’s possible HIV risk factors; the summary classification results from selecting, from the presumed hierarchical order of probability, the single risk factor most likely to have been responsible for acquiring HIV infection. Persons with > 1 reported risk factor are classified in the category listed first in the hierarchy. The exception is men who had sexual contact with other men and injected drugs; this group makes up a separate transmission category. Persons whose transmission category is classified as male-to-male sexual contact include men who have ever had sexual contact with other men, including men who have ever had sexual contact with men and with women. Persons whose transmission category is classified as heterosexual contact are persons who have ever had heterosexual contact with a person known to have, or to be at high risk for, HIV infection (e.g., a person who injects drugs).

**Fig 1:**
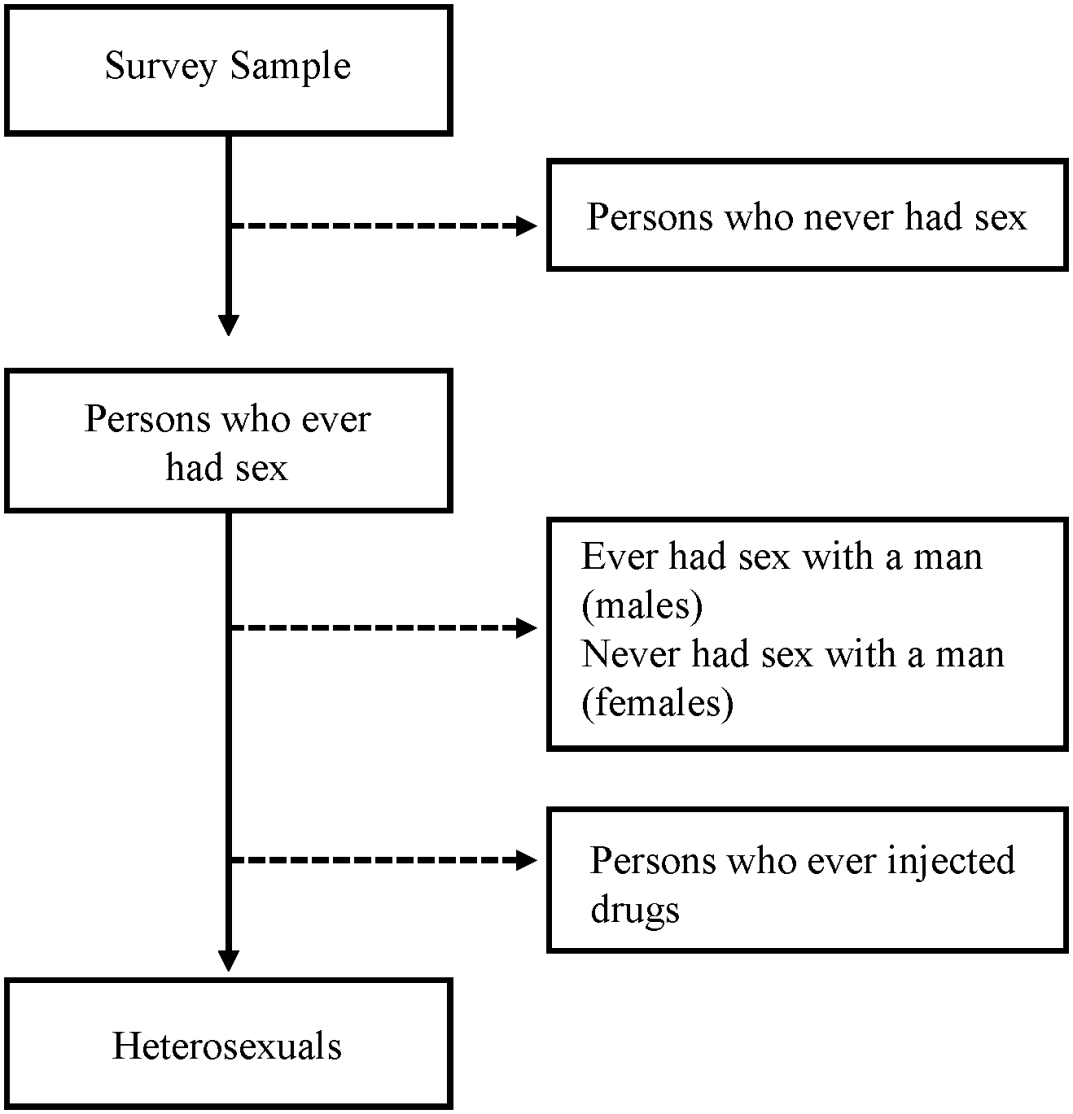
Definition of Heterosexual for Meta-Analysis.

Following the hierarchy used for transmission category, we calculated the proportion heterosexual with no other HIV risk behaviors from survey data (described below) by excluding the following: 1) those who reported never having had sex, 2) males who reported sex with another male, 3) males and females who ever injected drugs, and 4) females who only reported sex with female partners (i.e., never had sex with a man). The remaining proportion, excluding those with missing data, was considered heterosexual (Fig 1). Our choice to use lifetime (ever) behavior to define heterosexuals corresponded to the transmission category definition, which is behavior since 1977 [1].

### Data Sources for Calculating the Proportion of Heterosexuals in the U.S.

The three national population-based surveys included were the National Survey of Family Growth (NSFG, 2006–2010), the National Health and Nutrition Examination Survey (NHANES, 2009–2010), and the General Social Survey (GSS, 2010). These surveys and general question wording are described in Table 1; website addresses are provided for further information about the sampling methods, human subjects review, specific question wording, response rates, and weighting.

**Table 1:** Description of 3 national household surveys of the non-institutionalized population of the United States used in meta-analysis. CAPI = Computer-Assisted Personal Interview; ACASI = Audio, Computer-Assisted Self Interview

Survey Name	Population Surveyed	Sampling Method	Data used in meta-analysis	Interview method *	Question wording ^†^	Website
**General Social Survey (GSS)**	Persons aged ≥18 years, who spoke English or Spanish	Probability sample	Male and female respondents to the 2010 survey (aged 18–69 years).§	CAPI	Number of male/female sex partners since 18th birthday; Ever taken drugs by injection with a needle (not including drugs taken under a doctor’s orders).	http://www3.norc.org/GSS+Website/
**National Health and Nutrition Examination Survey (NHANES)**	Persons aged 12 to 69 years, who spoke English or Spanish	Stratified, multistage probability cluster design	Male and female respondents to the 2009–2010 sexual and drug use questionnaires (aged 15–69 years).¶	ACASI	Ever had oral, vaginal, or anal sex with an opposite-sex partner; Never used a needle to inject illegal drugs; Never had any type of sex with another man (male respondents only).	http://www.cdc.gov/nchs/nhanes.htm
**National Survey of Family Growth (NSFG)**	Persons aged 15–44 years who spoke English or Spanish	Multistage area probability sample	Male and female respondents to the 2006–2010 sexual and drug use questionnaires (aged 15–44 years).	ACASI	Ever had vaginal, oral, or anal sex with a male/female; Ever had oral or anal sex with a male (males only).	http://www.cdc.gov/nchs/nsfg.htm

* Interview method is for the sexual and drug use behavior questions.

^†^ Question wording includes all questions used to determine heterosexual (ever had sex with opposite sex partner, did not ever inject drugs, did not ever have sex with same-sex partner [males]). Note that for NSFG the questions on injection drug use were not used.

^§^ Analyses were limited to those aged 18–69 years to match the upper age limit of NHANES.

^¶^ Data were available for respondents aged 14–69 years. Analyses were limited to those aged 15–69 years to match the lower age limit of NSFG.

For NSFG and GSS, we determined the proportion of heterosexuals and the variance (standard error) using public use datasets. We obtained permissions to use restricted data for NHANES to include persons 15 and older as the public use dataset only includes persons aged 18 years and older. We used 15 years as the lower age limit for NHANES to match the lower age limit of NSFG. For GSS, we used 69 years as the upper age limit to match NHANES; the lower age limit for eligibility in GSS is 18 years. Because NSFG data on lifetime injection drug use were not available in the public use dataset, we used population proportions of persons who inject drugs from previous analyses [3] to adjust the estimated population proportion heterosexual for NSFG.

For each survey, we calculated the proportions of heterosexuals for the overall population. Stratified analyses were conducted by sex, race/ethnicity (non-Hispanic white, non-Hispanic black, Hispanic, and all others), and age group (15–24 years, 25–34 years, 35–44 years, 45–54 years, and 55–69 years) and for males and females by race/ethnicity and age group. For these analyses, we used SUDAAN software version 9.1 (RTI International, Research Triangle Park, NC) [5] to account for the complex sample designs. Differences in stratum-specific estimates were determined by non-overlapping confidence intervals.

### Meta-analysis for Estimating the Population Proportion of Heterosexuals

To combine the 3 distinct estimates into a combined measure, we applied a meta-analytic method that has recently been extended to survey data [6]. We first multiplied each survey estimate by a weight inversely proportionate to its variance, summed the weighted estimates across studies, and then divided by the sum of the weights.

The studies included for the meta-analysis were sufficiently homogeneous in terms of sampling methods, participants, and outcomes to provide a meaningful combined measure. All were national probability surveys designed to make inference to the U.S. household-based population, and collected self-reported data on sexual behavior. Despite these similarities, it is possible that differences in characteristics of the surveys, such as question wording, could result in heterogeneity. We selected random effects models for our analyses because the models assume the studies are a random sample [7], a type of inference that fits the purpose of our study which is to estimate the population proportion of heterosexuals. In our analysis, the estimates of the proportion heterosexual are not identical across surveys but rather have a distribution (under the random effects model assumption); the combined estimate describes the average of the survey estimates and the confidence interval provides an indication of the spread of the distribution of population proportion estimates of heterosexuals. The meta-analysis method developed by Rao et al [6] adds a between-studies variance term in deriving an overall estimate. Heterogeneity of estimates across surveys is indicated with the Q statistic [6] and Higgins' I^2^ index [8]. The Q statistic follows a chi-square distribution and assesses whether observed differences in results are compatible with chance alone. I^2^ describes the percentage of the variability in effect estimates that is due to heterogeneity rather than sampling error [9]. Values of the Q statistic indicated that the between-studies variance term was a statistically significant source of variability. Stratified analyses by sex and age allowed us to further address sources of heterogeneity across surveys.

We carried out all estimates per Rao’s method using Microsoft Excel (2007) and verified them using SAS Version 9.3 (SAS Institute, Cary, NC) [10]. We used the Comprehensive Meta-Analysis software version 2 (Biostat, Englewood, NJ) [11] to verify results and assess comparable patterns in the data.

### Method for Estimating the Numbers of Heterosexuals

We multiplied our derived estimates of the population proportion of heterosexuals by the population estimate from the Census Bureau for persons aged 13 years or older for the 50 states and District of Columbia [12] to obtain an estimated number of heterosexuals. The population proportions of heterosexuals in the age group 15–24 years were applied to the population aged 13–24 years and the population proportions of heterosexuals in the age group 55–69 years were applied to the population aged 55 years or older. Because persons in the youngest age group (13–14 years) are less likely to have had sex than those aged 15–24 years [13], this may result in an over-estimate of the number of heterosexuals in the youngest age group and result in an under-estimate of the rates of HIV infection. It should be noted, however, that persons aged 13–14 years make up a relatively small proportion of the entire age group (13–24 years) and thus the overall effect of over-estimating the number of heterosexuals is likely to be small.

### Method for Calculating HIV Disease Rates and Rate Ratios

We calculated HIV rates by dividing the estimated number of HIV cases attributed to heterosexual contact (numerator) by the estimated number of heterosexuals (denominator). Corresponding to measures included in annual HIV surveillance reports [1], we calculated two types of HIV rates: 1) diagnosis rates and 2) the rates of heterosexuals living with diagnosed HIV infection. For the numerators, we used HIV case surveillance data from all 50 states and the District of Columbia reported to CDC as of June 2014 for adults and adolescents (age 13 years or older at diagnosis) diagnosed with HIV infection in 2013 and for those living with diagnosed HIV infection as of December 2012.

For the denominators, we used the estimated number of heterosexuals. Denominators were calculated by multiplying census data by the population proportion of heterosexuals derived from the meta-analysis. We used 2013 and 2012 census data, respectively, to determine the number of heterosexuals for the HIV diagnosis rates and rates of living with diagnosed HIV infection.

We calculated rate ratios to compare rates by sex, race/ethnicity, and age. Males, whites, and the youngest age group (13–24 years) served as the reference groups, respectively.

### Analysis of “High-risk Heterosexual”

This study’s objective was to provide an estimate of the total number of heterosexuals in the U.S. and the HIV prevalence rate among heterosexuals, without accounting for the level of risk in their sexual behavior. Defining “high-risk heterosexuals” is complex [14]. Many people do not know the HIV status of their partners [14] and thus could not reliably report this risk. Other risk behaviors, such as high number of sex partners, are not sensitive enough to capture those who may have had only a single—albeit HIV-infected—partner. To calculate disease rates, the persons included in the numerator (HIV infection attributable to heterosexual contact) also must be included in the denominator (at risk for heterosexually acquired HIV infection). Definitions of “high-risk heterosexual” can exclude some HIV-infected persons in the denominator. Conversely, broader markers for HIV risk, such as any condomless sex, are not very specific and would not exclude those at low risk for infection in a population with comparatively lower rates of HIV prevalence.

We calculated the population proportion high-risk heterosexual and used that to calculate HIV prevalence. For the population proportion high-risk heterosexual, we used the data from NSFG [15] which reported approximately 5.6% of the general U.S. population age 15–44 years had a lifetime HIV-related sexual risk behavior, such as having 5 or more sex partners, exchanging sex for money or drugs, having a male sex partner who had sex with men or injected drugs, or had a sex partner who was HIV-positive. Subtracting the proportion of males that had male-male sex (2.1%) [15] gives a resulting estimate of 4.5% of the population being high-risk heterosexual.

Similar to our main analyses, we calculated HIV rates among high-risk heterosexuals by dividing the estimated number of HIV cases attributed to heterosexual contact (numerator) by the estimated number of high-risk heterosexuals (denominator). Denominators were calculated by multiplying census data by the 4.5% population proportion of high-risk heterosexuals derived from the NSFG data. The high-risk subset of the heterosexual population yields a smaller population denominator, thus the HIV prevalence of heterosexually acquired HIV infection calculated with the high-risk heterosexual estimate represents an upper bound estimate of HIV prevalence.

## Results

### Proportion of the Population and Number Estimated to be Heterosexual in the U.S.

Table 2 shows the estimated population proportion of lifetime heterosexuals overall and for males and females for each population-based survey and the combined estimates from the meta-analysis. The overall combined estimate was 86.7% (95% confidence interval [CI]: 84.1%-89.3%). As noted, Q statistics and I^2^ indicated heterogeneity of results across the surveys (I^2^ = 91.6; Q = 23.7, p < 0.001). The combined estimate for males was 84.1% (CI: 81.2%-86.9%) and for females was 89.4% (95% CI: 86.9%-91.8%).

**Table 2:** Estimated proportion of heterosexual persons in the United States, by survey and combined by meta-analysis.

Population	Survey	% Heterosexual	95% CI	
Males*				
	GSS	85.0	81.7	87.7
	NHANES	85.8	83.9	87.7
	NSFG	81.6	79.8	83.4
	**Combined estimate**	**84.1**	**81.2**	**86.9**
Females ^†^				
	GSS	88.5	86.1	90.6
	NHANES	91.5	90.2	92.7
	NSFG	87.9	86.5	89.4
	**Combined estimate**	**89.4**	**86.9**	**91.8**
Total ^§^				
	GSS	86.9	85.0	88.6
	NHANES	88.6	87.2	90.0
	NSFG	84.8	84.0	85.5
	**Combined estimate**	**86.7**	**84.1**	**89.3**

* I^2^ = 81.1; Q = 10.6, p = 0.005

^†^ I^2^ = 88.1; Q = 16.8, p <0.001

^§^ I^2^ = 91.6; Q = 23.7, p < 0.001. CI = confidence interval. GSS = General Social Survey (2010); NHANES = National Health and Nutrition Examination Survey (2009–2010); NSFG = National Survey of Family Growth (2006–2010). See Table 1 for description of each survey.

Applying these proportions to the U.S. population age 13 years or older for 2013, we estimate that approximately 228,402,110 adults and adolescents are heterosexuals, with an estimated range, based on the confidence intervals for the population proportion estimate, from 221,593,250 to 235,210,969 persons; using the sex-specific proportions represents an estimated 108,187,901 heterosexual males (range: 104,486,382–111,889,420) and 120,368,338 heterosexual females (range: 117,061,991–123,674,686). The proportion heterosexual among males is lower than among females in part because of exclusions for MSM and for PWID (the proportion PWID is higher among males than females) [3].

We calculated population proportion estimates for male and female heterosexuals by race/ethnicity and by age group (Table 3). The population proportion of heterosexuals did not differ significantly by race/ethnicity among males or females. The population proportion of heterosexuals was lowest among both males and females aged 15–24 years, and was not significantly different among those aged 25–69 years. Estimates for females for the age categories of 35–44 years, 45–54 years, and 55–69 years and the overall estimate for those aged 55–69 years had a relative standard error (RSE) of 30%-49%. In general, estimates with a RSE of 30% or greater are subject to high sampling error and are recommended to be used with caution. Thus, the specified estimates with RSE ≥30% and the resulting rates presented in Table 3 should be interpreted with caution.

**Table 3:** Estimated proportion of heterosexual persons in the United States, by sex, race/ethnicity, and age group--meta-analysis of 3 national surveys*. CI = confidence interval.

	Males			Females			Total		
	% Heterosexual	95% CI		% Heterosexual	95% CI		% Heterosexual	95% CI	
**Race/Ethnicity**									
White	84.8	80.4	89.2	89.8	86.4	93.3	87.3	84.1	90.5
Black/African American	80.6	74.3	87.0	89.5	87.8	91.1	86.6	84.7	88.5
Hispanic/Latino	85.4	83.4	87.3	88.9	87.6	90.1	87.0	86.0	88.0
Other	80.2	75.5	85.0	85.9	82.4	89.3	82.4	79.3	85.6
**Age Group (years)**									
15–24	70.2	65.4	74.9	72.1	68.8	75.4	72.2	67.4	77.0
25–34	88.9	87.2	90.7	94.7	91.7	97.8	91.7	89.8	93.7
35–44	89.1	84.9	93.4	95.3	92.4	98.2†	92.1	88.9	95.2
45–54	88.0	85.6	90.5	91.8	84.5	99.0†	89.7	85.1	94.4
55–69	89.4	84.7	94.2	94.7	89.7	99.6†	91.9	87.1	96.7
**Total**	**84.1**	**81.2**	**86.9**	**89.4**	**86.9**	**91.8**	**86.7**	**84.1**	**89.3**

*Surveys used in the meta-analysis: General Social Survey (2010); NHANES = National Health and Nutrition Examination Survey (2009–2010); NSFG = National Survey of Family Growth (2006–2010). See Table 1 for description of each survey.

^†^ Relative Standard Error (RSE) = 30–49%.

### HIV Disease Rates and Rate Ratios among Heterosexuals in the U.S.

Rates of diagnosis of HIV infection among heterosexuals and rates of heterosexuals living with diagnosed HIV infection are presented in Tables 4 and 5, respectively. The rate of diagnosis of HIV infection was 5.2 per 100,000 heterosexuals (CI: 5.1–5.4); the rate of heterosexuals living with diagnosed HIV infection was 104 per 100,000 heterosexuals (CI: 101–108), or 0.1%. The rates for females were higher than those for males for diagnosis rate (rate ratio: 1.9, CI: 1.7–2.0) and for rate of persons living with diagnosed HIV infection (rate ratio: 2.0, CI: 1.9–2.2).

**Table 4:** Diagnoses of HIV infection among adult and adolescent heterosexuals, by selected characteristics—United States, 2013. Note. Data include persons age 13 years and older with a diagnosis of HIV infection regardless of stage of disease at diagnosis. CI = confidence interval

			No. *	Rate†	95% CI		Rate Ratio†	95% CI	
**Males**			**3,887**	**3.6**	**3.5**	**3.7**	**1.0**		
	**Race/ethnicity**								
		White	530	0.8	0.7	0.8	1.0		
		Black	2,493	20.6	19.1	22.4	27.5	24.2	31.4
		Hispanic/Latino^§^	718	4.1	4.0	4.2	5.4	5.0	5.8
		Other^¶^	146	1.9	1.8	2.0	2.6	2.3	2.9
	**Age at diagnosis**								
		13–24	294	1.6	1.5	1.7	1.0		
		25–34	787	4.1	4.0	4.2	2.6	2.4	2.8
		35–44	926	5.2	4.9	5.4	3.3	2.9	3.7
		45–54	1,064	5.6	5.4	5.8	3.6	3.3	3.9
		55+	817	2.4	2.2	2.5	1.5	1.3	1.7
**Females**			**8,031**	**6.7**	**6.5**	**6.9**	**1.9**	**1.7**	**2.0**
	**Race/ethnicity**								
		White	1,174	1.5	1.4	1.6	1.0		
		Black	5,268	34.9	34.3	35.6	23.3	22.0	24.6
		Hispanic/Latino^§^	1,232	6.9	6.8	7.0	4.6	4.3	4.8
		Other^¶^	356	4.0	3.8	4.1	2.6	2.4	2.9
	**Age at diagnosis**								
		13–24	1,110	6.0	5.8	6.3	1.0		
		25–34	2,060	10.2	9.9	10.6	1.7	1.6	1.8
		35–44**	1,936	10.0	9.7	10.3	1.7	1.5	1.8
		45–54**	1,792	8.8	8.1	9.6	1.5	1.3	1.6
		55+**	1,132	2.6	2.5	2.8	0.4	0.4	0.5
**Total**			**11,918**	**5.2**	**5.1**	**5.4**			
	**Race/ethnicity**								
		White	1,704	1.1	1.1	1.2	1.0		
		Black	7,761	28.1	27.5	28.8	24.6	23.2	26.1
		Hispanic/Latino^§^	1,951	5.5	5.4	5.5	4.8	4.6	5.0
		Other^¶^	502	3.1	3.0	3.2	2.7	2.5	2.9
	**Age at diagnosis**								
		13–24	1,404	3.7	3.5	4.0	1.0		
		25–34	2,847	7.2	7.1	7.4	1.9	1.8	2.1
		35–44	2,862	7.7	7.4	8.0	2.1	1.9	2.3
		45–54	2,855	7.3	6.9	7.7	2.0	1.7	2.2
		55+**	1,949	2.5	2.4	2.7	0.7	0.6	0.8

*Number of cases attributable to heterosexual contact, statistically adjusted to account for reporting delays and missing risk factor information, but not for incomplete reporting.

^†^Per 100,000 heterosexuals.

^§^ Hispanics/Latinos may be of any race.

^¶^ Other race includes American Indian/Alaska Native, Native Hawaiian/Other Pacific Islander, unknown race/ethnicity, and multiple races.

** Relative standard error >30% for meta-analysis estimate of the population proportion heterosexual for this group.

**Table 5:** Adult and adolescent heterosexuals living with diagnosed HIV infection- United States, 2012. Note. Data include persons age 13 years and older with a diagnosis of HIV infection regardless of stage of disease at diagnosis. CI = confidence interval

			No. *	Rate†	95% CI		Rate Ratio†	95% CI	
**Males**			**72,482**	**67.7**	**65.4**	**70.0**	**1.0**		
	**Race/ethnicity**								
		White	9,257	13.1	12.5	13.8	1.0		
		Black	46,795	392.3	363.7	425.7	29.9	26.3	34.2
		Hispanic/Latino^§^	13,600	78.7	76.9	80.5	6.0	5.6	6.5
		Other^¶^	2,830	38.5	36.4	40.9	2.9	2.6	3.3
	**Age at diagnosis**								
		13–24	974	5.2	4.9	5.6	1.0		
		25–34	6,666	35.1	34.5	35.8	6.8	6.2	7.4
		35–44	16,627	92.5	88.3	97.1	17.8	15.9	20.0
		45–54	26,824	139.7	135.9	143.7	26.9	24.4	29.6
		55+	21,390	63.9	60.7	67.5	12.3	10.9	13.9
**Females**			**163,995**	**137.4**	**133.7**	**141.3**	**2.0**	**1.9**	**2.2**
	**Race/ethnicity**								
		White	25,308	32.4	31.2	33.7	1.0		
		Black	103,953	697.0	684.5	709.9	21.5	20.3	22.8
		Hispanic/Latino^§^	27,425	156.7	154.6	158.9	4.8	4.6	5.1
		Other^¶^	7,270	83.5	80.3	87.0	2.6	2.4	2.8
	**Age at diagnosis**								
		13–24	5,379	29.3	28.0	30.7	1.0		
		25–34	27,232	137.1	132.8	141.7	4.7	4.3	5.0
		35–44**	48,389	249.6	242.1	257.4	8.5	7.9	9.2
		45–54**	50,959	247.2	229.0	268.5	8.4	7.5	9.6
		55+**	31,996	76.3	72.5	80.5	2.6	2.4	2.9
**Total**			**236,437**	**104.4**	**101.4**	**107.7**			
	**Race/ethnicity**								
		White	34,565	23.2	22.4	24.1	1.0		
		Black	150,749	553.4	541.4	566.0	23.8	22.4	25.2
		Hispanic/Latino^§^	41,024	118.0	116.7	119.4	5.1	4.8	5.3
		Other^¶^	10,099	63.5	61.2	66.0	2.7	2.5	2.9
	**Age at diagnosis**								
		13–24	6,353	16.9	15.8	18.1	1.0		
		25–34	33,899	87.4	85.5	89.3	5.2	4.7	5.6
		35–44	65,016	174.3	168.6	180.5	10.3	9.3	11.4
		45–54	77,783	195.8	186.1	206.6	11.6	10.3	13.1
		55+**	53,387	71.1	67.6	75.0	4.2	3.7	4.7

*Number of cases attributable to heterosexual contact, statistically adjusted to account for reporting delays and missing risk factor information, but not for incomplete reporting.

^†^Per 100,000 heterosexuals.

^§^ Hispanics/Latinos may be of any race.

^¶^ Other race includes American Indian/Alaska Native, Native Hawaiian/Other Pacific Islander, unknown race/ethnicity, and multiple races.

** Relative standard error >30% for meta-analysis estimate of the population proportion heterosexual for this group.

The rate ratios revealed disparities by race/ethnicity and by age. Comparing black males to white males, the estimated rate of diagnoses of HIV infection was 24–31 times as high (Table 4) and the estimated rate of living with diagnosed HIV infection was 26–34 times as high (Table 5); comparing Hispanic/Latino to white males, these rates were approximately 5–6 times as high, for both rates (Tables 4 and 5). Comparing black females to white females, the estimated rate of diagnosis of HIV infection was 22–25 times as high (Table 4) and the estimated rate of living with diagnosed HIV infection was 20–23 times as high (Table 5); comparing Hispanic/Latino to white females, these rates were approximately 4–5 times as high for both measures (Tables 4 and 5). Among males, the rate of diagnoses and living with diagnosed HIV was higher among older age groups than those 13–24 years. The population proportion heterosexual among females stratified by age group had some RSE ≥30%, and thus rates and rate ratios should be interpreted with caution (Tables 4 and 5).

### HIV Prevalence among “High-Risk Heterosexuals”

The HIV prevalence among high-risk heterosexuals was 2.0% (data not shown), or 20 times as high as our overall prevalence of 0.1%. Considering only this high-risk subset of the heterosexual population yields a smaller population denominator (4.5% vs. 87%) and thus a higher HIV prevalence than our result among all heterosexuals.

## Discussion

Using data from three national population-based U.S. surveys, we estimated that heterosexuals comprised 86.7% (CI: 84.1%-89.3%) of the U.S. adult and adolescent population; 84% among males (95% CI: 81.2%-86.9%) and 89% among females (95% CI: 86.9%-91.8%). These proportions are somewhat lower than self-reported sexual orientation from a national probability survey on sexual behavior, which reported that >90% of adults and adolescents were heterosexual [16]. The difference is not surprising as heterosexuals defined here were based on sexual behavior, not sexual orientation, and excluded those who engaged in other HIV risk behaviors (e.g., drug use or male-male sexual behavior).

Our estimates also quantified the recognized disparity of HIV disease rates among black and Hispanic/Latino male and female heterosexuals when compared with white male and female heterosexuals. Rates were more than 20 times as high among blacks as compared to whites and five times as high among Hispanics/Latinos as compared to whites. Additionally, the need for risk group-specific rates is illustrated by comparing the differences in the population-based rates found in HIV surveillance reports with our rates among heterosexuals only. Our data give a clearer picture of rates and disparities among heterosexual men by race/ethnicity than rates for all men, which are affected by cases attributable to male-male sexual contact (comprising 79% of diagnoses among men) [1]. For example, the diagnosis rate ratios comparing black and Hispanic males to white males are considerably higher among heterosexual men (27.5 and 5.4 per 100,000 population, respectively) than men overall (7.7 and 3.0, respectively [1]). Because a high proportion of cases among women are attributed to heterosexual contact, the rates for female heterosexuals look very similar to population-based rates for females. [1]

Our results are subject to several limitations. While the study designs of the 3 national surveys are robust, they have small numbers of participants reporting male-male sex or injection drug use. The limitations of these surveys for measuring these behaviors are discussed in detail elsewhere [2–3]. Under-reporting of these behaviors would result in over-estimation of the population proportion heterosexual; however this bias should be mitigated in part by use of ACASI for two of the surveys included in our analysis. A second limitation is the degree of heterogeneity among surveys. Although all surveys are population-based, the sampling methods, age range, and question wording vary across surveys. We used random-effects models to account for variance beyond sampling errors. Third, sample sizes for the surveys when stratified by age group likely contributed to the large RSEs noted for females and overall; GSS has a relatively small sample size overall and NSFG has an upper age limit of 44 years. A fourth limitation, as noted above, is that the rates among those aged 13–24 years and 55 years or older may be under-estimates given that the meta-analysis was limited to those aged 15–69 years. While the use of a separate estimate of the proportion of persons who inject drugs to adjust the NSFG data may have biased the NSFG estimate of heterosexuals, the NSFG estimates of heterosexuals were not considerably different from the other two surveys and thus any biases are likely small. Other limitations are inherent from the surveillance data used in the rate calculations [1].

Given the potential factors affecting the data in the 3 surveys and the surveillance data, the population estimates and disease rates should be presented with acknowledgement of their limitations and interpreted in the context of the confidence intervals presented. Wider confidence intervals for some groups indicate less precision in the estimates, particularly for the subgroup analyses.

Calculating an HIV prevalence rate specifically for high-risk heterosexuals is complicated by the lack of consistency between the definition of high-risk heterosexuals used to establish the denominator and the definition used to establish the number of persons living with HIV infection attributable to heterosexual contact. Therefore, our results should be interpreted with caution; the HIV rates reported here among all heterosexuals underestimate rates for high-risk heterosexuals given the inclusion of those with lower risk in the denominator. Assuming all persons in the numerator did meet a definition of high risk, the estimated prevalence could be as high as 2%.

In addition to high-risk sexual behaviors, socioeconomic factors and other social determinants of health may also contribute to higher rates of HIV infection in some groups of heterosexuals. The National HIV Behavioral Surveillance System used a definition of risk for heterosexually acquired HIV infection that focused on income and education and recruited within networks of persons living in areas of high HIV prevalence. In these surveys, HIV prevalence among NHBS participants was approximately 2% [17].

Estimating the population proportion of heterosexuals allowed calculation of rates of HIV infection and allows for examining disparities within groups. Trends from population-based surveys will be monitored as part of CDC’s behavioral surveillance analyses, and the meta-analysis can be updated as new data emerge. Rates can be calculated on an annual basis with the most recent surveillance data. Other disease metrics can be used to calculate rates, such as HIV incidence [18] or national HIV prevalence estimates [19], which include persons with undiagnosed HIV infection. Our estimates may not be well suited for calculating disease rates at the state or local level as the population sizes of MSM and PWID—and therefore heterosexuals—vary across the U.S. [20–21] and by urbanicity [22].

The best available data must be used to guide decision-making for HIV prevention at the national, state, and local levels. The estimate of the number of heterosexuals in the U.S. and burden of HIV infection among them can be particularly important for planning and evaluating programs serving disproportionately affected populations and addressing health inequities. The estimate of the number of heterosexuals in the U.S. and resulting HIV rates are important additions to cost effectiveness and other data used to make critical decisions about resources for prevention of HIV infection.

## Supporting Information

S1 Table PRISMA Checklist. (DOC)
